# Melodic Intonation Therapy in Post-Stroke Non-Fluent Aphasia and Its Effects on Brain Plasticity

**DOI:** 10.3390/jcm11123503

**Published:** 2022-06-17

**Authors:** Natalia García-Casares, Amanda Barros-Cano, Juan A. García-Arnés

**Affiliations:** 1 School of Medicine, University of Málaga, 29010 Málaga, Spain; arnes@uma.es; 2 Unidad de Neurología Clínica, Centro de Investigaciones Médico-Sanitarias (CIMES), Campus de Teatinos, University of Málaga, 29010 Málaga, Spain; 3 Instituto de Investigación Biomédica de Málaga-IBIMA, 29010 Málaga, Spain

**Keywords:** melodic intonation therapy, neuroimaging, functional magnetic resonance (fMRI), diffusion tensor imaging (DTI), positron emission tomography (PET), spectroscopy positron emission tomography (SPECT)

## Abstract

Melodic Intonation Therapy (MIT) is one of the most well-known therapies for the rehabilitation of speech in patients with non-fluent aphasia and which is thought to promote right-hemisphere involvement in language processing. This review focuses on the study of language lateralization and/or neuroplastic reorganization with neuroimaging and/or neurophysiological techniques in non-fluent aphasic patients post-stroke during or after MIT. A systematic search was carried out according to the Preferred Reporting Items for Systematic reviews and Meta-Analyses (PRISMA) in databases (PubMed, Scopus, EMBASE, Dialnet, Web of Science, Cochrane) with the keywords melodic intonation therapy, neuroimaging, functional magnetic resonance, and positron emission tomography and the boolean operators AND and OR. Articles including patients of all ages and either sex with any type of aphasia post-stroke and in any language, which studied language lateralization and/or neuroplastic reorganization after or during MIT were included. Articles which did not achieve the objectives, revisions and conferences were excluded. Different results were obtained from the 16 studies included in the review: predominantly greater activation of the right hemisphere but also of the left hemisphere or both. MIT is an effective therapy to rehabilitate non-fluent aphasic patients post-stroke. It involves different neurobiological mechanisms and depends on multiple individual factors. Studies with larger samples are necessary.

## 1. Introduction

Aphasia is a common symptom following a stroke, affecting 21–40% of patients. The recovery of language functions depends mostly on the location and size of the infarction, severity of the initial neurological deficits, and the individual characteristics of the patient. At present, many post-stroke aphasia treatment strategies are available, and rehabilitation should be adjusted to the individual needs of the patients. Speech and language therapy are the most used, but other options include cognitive neurorehabilitation, telerehabilitation, and computer-based management [1]. Moreover, in some studies, drug therapy and transcranial direct stimulation have proven to play an important role in the treatment of post-stroke aphasia [1,2].

Melodic intonation therapy (MIT) was introduced in 1973 by Albert, Sparks, and Helm. This therapy is based on the observation that people with aphasia are able to sing familiar songs [3,4]. The candidates who are considered good responders to this therapy meet the following characteristics: they suffer non-fluent aphasia (Broca) post-stroke with impaired repetition and slow, poorly articulated speech. Moreover, their brain lesions include Broca’s area (cortical and deep) or undercut Broca’s area. However, their auditory comprehension and emotional stability, Wernicke’s area, and also the right hemisphere should be intact [5,6].

MIT contains two unique components: intonation and rhythm. The main objective of this therapy is to restore language output by learning a new way of speaking through melodic intonation. First, spoken words and phrases are intoned using two pitches: a high pitch for the stressed syllables and a low pitch for the unstressed syllables, which are usually separated by a third or fourth (usually of a minor third). Second, the rhythmic patterns are formed between 4 and 8 notes, and the tempo is lengthened. Moreover, it is an intensive treatment since the patient receives frequent sessions (at least 3 weeks), totaling 1.5 h/day, until the three levels have been completed. Thus, the clinician asks the patient to produce everyday life sentences in a singing-like manner, which exaggerates the normal prosody while tapping with the left hand on each syllable. Furthermore, there are three levels of difficulty: initially, the patient intones in unison with the therapist, and gradually, the patient progresses to more autonomous production. Finally, they suspend the rhythm and intonation of their speech. Each level consists of 20 items which are words or social phrases presented with visual cues [7,8,9]. MIT is very well-known nowadays and has been modified by clinicians and even been adapted to other languages and cultures. In fact, other versions such as TMR, which is the French version of MIT, teach the patient to use intonation as a facilitating technique in different situations [10]. Palliative versions, however, are for patients who have a very impaired language function and just help the patients to learn a set of limited sentences for basic communication in daily life [7].

The main hypothesis is that the components of MIT (intonation and rhythm) promote the use of the right hemisphere in order to compensate the damage in the language regions of the left hemisphere. However, this hypothesis remains under debate, as there are two points of view: MIT promotes upregulation of neural activity in the right hemisphere or in the perilesional left hemisphere. For this reason, the consensus is that there are two routes of recovery. On the one hand, in patients with small lesions, there is a greater involvement of the left-hemisphere peri-lesional pathways, and a variable right-hemisphere involvement. On the other hand, in patients with large left-hemisphere lesions (including most of the language regions), there is a higher involvement of homologous right-hemisphere language regions [7,8].

Previous studies have attempted to discern the different neurobiological mechanisms which promote language recovery and neuroplastic reorganization through MIT therapy. First, a recent review demonstrated its effectiveness in language improvement [11]. Second, in other reviews, the different neurobiological and cognitive mechanisms of MIT were discussed. For example, Zumbansen et al. [7] undertook a critical review of the literature of MIT and included studies with neuroimaging techniques in order to study the role of the right hemisphere. However, the results were very heterogeneous: in some cases, the MIT original hypothesis was supported, whereas, in others, the left hemisphere has a dominant role or even both hemispheres. Likewise, Merret et al. [8] reviewed the evidence for the different mechanisms explaining the effectiveness of MIT: neuroplastic reorganization of language function, activation of the mirror neuron system, motivation, and mood and the shared or specific features of music and language. They conclude that the interaction of these mechanisms has an additive effect and all contribute to language recovery, making MIT more efficient than other therapies. Finally, Norton et al. [9] describe MIT therapy and the effects of its different components (intonation, left hand tapping, etc.) in neuroplastic reorganization. Nonetheless, these reviews [7,8,9] all have certain limitations because of the small sample sizes and failed to reach any general conclusions. In fact, they concluded that more research would be necessary to understand the neural reorganization and processes of MIT.

For this reason, the main objective of this systematic review is to include all the studies with any neuroimaging/neurophysiological techniques to study language lateralization and neuronal reorganization during or after rehabilitation with MIT in post-stroke aphasic patients in order to continue the previous research and to obtain further conclusions and information about the role of MIT in neuroplastic reorganization.

## 2. Materials and Methods

This systematic review is reported according to the Preferred Reported Items for Systematic reviews and Meta-Analyses (PRISMA) [12]. The systematic review was registered in the database PROSPERO, https://www.crd.york.ac.uk/prospero/#searchadvanced (accessed on 10 June 2022) where it can be accessed.

Our PICO (population, intervention, comparator, outcome) question was: what are the neurobiological mechanisms of MIT which promote language recovery and neuroplastic reorganization in patients with non-fluent aphasia post-stroke by using neurophysiological or neuroimaging tests?

### 2.1. Search Strategy

The following databases were searched: PUBMED, SCOPUS, EMBASE, DIALNET, Web of Science, Cochrane. In addition, we also performed a manual search of reference lists in other prior systematic reviews on the same topic to identify further potentially eligible studies. The terms used in combination for the search were: melodic intonation therapy, neuroimaging, imaging, MRI, fMRI, MEG, PET, SPECT; and the boolean operators AND and OR. The last search was performed on 10 January 2022.

### 2.2. Inclusion Criteria

All articles about the neural reorganization and language lateralization which included neuroimaging/neurophysiological techniques were included. Studies in any language with participants of all ages, either sex, with any type of aphasia post-stroke were included. Moreover, articles which included participants who had previously received language rehabilitation with a different therapy from MIT, series of cases and unique cases were admitted. The literature ranges from MIT development in 1973 to nowadays.

### 2.3. Exclusion Criteria

Revisions, letters to the editor, and articles/cases which discussed neuroplastic reorganization with MIT but did not include neuroimaging or neurophysiological techniques were excluded. In addition, we also excluded studies with healthy participants (non-aphasic patients) and which used other rhythmic or intoned therapies which were not exactly MIT. Studies that did not achieve the objectives of this revision were also excluded. 

### 2.4. Data Collection and Analysis

**Figure 1:**
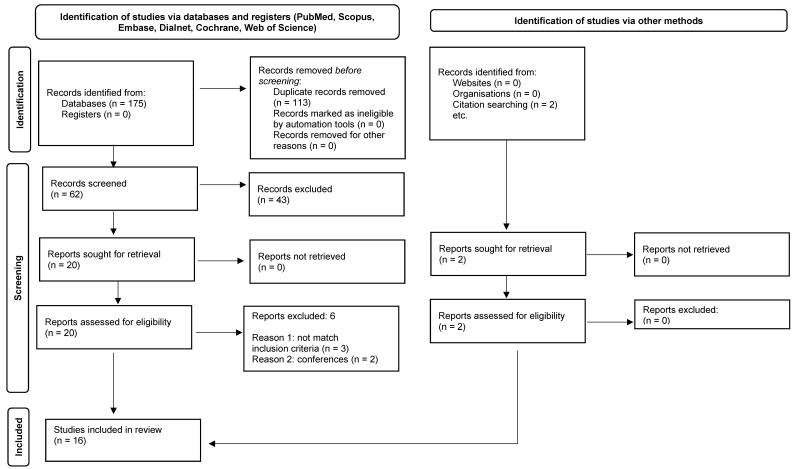
Flowchart of the search strategy.

A total of 175 records were identified via database search. After removing duplicates, 62 records were screened, leaving 20 full text-articles assessed for eligibility. Two articles were excluded as they were conferences [13,14], one article was excluded since it did not include any neuroimaging [15], and two others because they were revisions [16,17]. Moreover, two additional studies were obtained reviewing the reference lists of included articles and related reviews, which were also included [18,19]. Figure 1 shows the PRISMA 2020 flow diagram of study selection.

### 2.5. Quality Assessment 

The quality of the included studies was evaluated using the PEDro scale, which includes 11 criteria: eligibility criteria specified, allocation, concealed allocation, description of the condition being treated, blinding, key outcomes, and statistical comparisons between groups [20]. Table 1 contains the points obtained on each of these criteria of all the studies included in the review. The two authors reviewed independently each of the articles selected. Disagreements were resolved by consensus.

## 3. Results

### 3.1. Study Characteristics

Table 2 contains the 16 articles that proved eligible, arranged in chronological order. The following information has been included: authors and date of publication, type of study, intervention characteristics, aphasia type of the participants, imaging/neurophysiological techniques, imaging paradigm, involvement of the right or left hemisphere, and language benefits.

According to the design, six records were a series of cases [21,22,23,24,25,26,27] and five were studies with a unique case [18,19,28,29,30]. Two articles compared MIT with another therapy [31,32] and another two studies with a group of untreated participants [27,33]. In these four studies, a greater improvement in language function was demonstrated in the MIT-treated group. 

In all the studies, all the participants suffered moderate–severe chronic Broca’s aphasia after a stroke, except in four studies where patients had subacute [18,21], mixed [23,24,25], or Wernicke’s aphasia [25]. 

FMRI was used in six studies [18,21,27,29,31,34]. Three articles included magnetic resonance imaging (MRI) with diffusion tensor imaging (DTI) [26,32,33]. In addition, both functional magnetic resonance imaging (FMRI) and MRI(DTI) were used in two studies [22,30]. Brain activity was observed in different situations: silence, passive listening, humming, speaking, singing, naming, reading, and repetition of normal/intoned words. Positron emission tomography (PET) was used in one study to measure cerebral blood flow during rest, hearing, repetition of simple words, and MIT-loaded words [24]. Magnetoencephalography was used in another study to examine cerebral activity during an action-naming task [23]. Finally, Single-Photon Emission Computed Tomography (SPECT) was used in three studies to observe neuronal reorganization after MIT [28], language activation with simple repetition and MIT [25], and brain activity during rest, ordinary repetition, and MIT [19].

In the majority of articles, language lateralization, and neuroplastic reorganization were studied comparing the results of language tests and imaging techniques before and after MIT rehabilitation. Nonetheless, in one article [24], only post-treatment data were reported, and in two studies [19,25] only pre-treatment data was available.

### 3.2. Hemisphere Involvement

According to the main hypothesis of MIT, a few articles found a higher right hemisphere involvement. In five studies, white matter tracts in the right hemisphere were studied after MIT therapy and the following results were obtained: an increase in the number of fibers in the right arcuate and uncinate fasciculus [31] and an increase in FA values (fractional anisotropy) in the right superior longitudinal fasciculus [32]. In contrast, a decrement in FA values in white matter tracts underlying the pars opercularis and pars triangularis of the inferior frontal gyrus, the right posterior superior temporal gyrus, and the right posterior cingulum was observed in another article [33]. Moreover, in four studies [22,29,30,31], brain behavior was studied using fMRI after MIT, showing a higher right hemisphere activity, mainly in the right frontal lobe (inferior frontal gyrus), temporal lobe, occipital lobe, and cerebellum.

Nevertheless, in some articles, an increase in left hemisphere activity, especially in the frontal and temporal lobes involving Broca’s, Wernicke’s, and prefrontal areas was found, concerning a decrease in the homologous right hemisphere regions [23,24]. This was also evidenced in a patient with subacute aphasia [18]. Finally in two cases, after the application of repetitive transcranial stimulation (rTMS) in Broca’s right area, an increase in left hemisphere involvement was observed in the participant who was a good responder to MIT, whereas the poor responder showed an activation of both hemispheres [27]. 

Finally, several articles reported an involvement of both hemispheres [19,21,25,28,34]. In the cases that used SPECT to measure regional cerebral blood flow, an increase in perfusion was observed, mainly in the fronto-temporal regions in the left and also right hemisphere [19,25,28]. Merrett et al. [34] evidenced a deactivation of the peri-lesional areas in the left hemisphere concerning an activation of right frontal and bilateral auditory regions. Van de Sandt-Koenderman et al. [21] evaluated language lateralization in subacute and chronic aphasic patients, with the following results: a higher right lateralization was observed in the subacute cases and a greater left lateralization in the chronic cases. 

### 3.3. Clinical Benefit

Most patients included in the studies showed an amelioration of speech outcomes after MIT therapy. A greater improvement with this therapy was demonstrated even in the studies which compared MIT with conventional speech therapy [31,32] or with an untreated patient group [27,33]. However, in a few studies, the patients included did not benefit from MIT therapy [23,25,27,34].

**Table 1:** Points Obtained in Each Criterion of the PEDro Scale of the Studies Included in the Review.

Authors & Date of Publication	Criterion 1	Criterion 2	Criterion 3	Criterion 4	Criterion 5	Criterion 6	Criterion 7	Criterion 8	Criterion 9	Criterion 10	Criterion 11	TOTAL
Martzoukou et al. [28], 2021	1	0	0	1	0	0	0	1	1	0	0	4
Merrett et al. [34], 2018	1	0	0	1	0	0	0	1	1	0	0	4
Yang et al. [32], 2018	1	0	0	1	0	0	0	1	1	0	0	4
Sandt-koenderman et al. [21], 2016	1	0	0	1	0	0	0	1	1	0	0	4
Tabei et al. [29], 2016	1	0	0	1	0	0	0	1	1	0	0	4
Wan et al. [33], 2014	1	0	0	1	0	0	0	1	1	1	1	6
Al-Janabi et al. [27], 2014	1	0	1	1	1	1	0	1	1	1	1	9
Zipse et al. [30], 2012	1	0	0	0	0	0	0	1	1	0	0	3
Schlaug et al. [22], 2010	0	0	0	0	0	0	0	1	1	0	0	2
Sandt-Koenderman et al. [18], 2010	1	0	0	0	0	0	0	1	1	0	0	3
Breier et al. [23], 2009	1	0	0	0	0	0	0	1	1	0	0	3
Schlaug et al. [26], 2009	1	0	0	1	0	0	0	1	1	1	1	6
Schlaug et al. [31], 2008	1	1	0	1	0	0	0	1	1	1	1	7
Belin et al. [24], 1996	1	1	0	1	0	0	0	1	1	1	1	7
Laine et al. [25],1994	1	0	0	0	0	0	0	0	1	0	0	2
Christensen et al. [19], 1989	1	0	0	0	0	0	0	0	1	0	0	2

**Table 2:** Characteristics of the Studies Included in the Review.

Authors & Date of Publication	Design Study and Group	Intervention Characteristics	Participant Characteristics and Aphasia type	Imaging/Neurophysiological Techniques	Imaging Paradigm	Involvement of LH and RH	Language Benefits
Martzoukou et al. [28], 2021	N = 1 MIT in Greek	36 sessions in 12 weeks (30–40 min, 3 days/week)	Age: 64 male Ischemic stroke Severe chronic non-fluent aphasia	SPECT (99 mTc-HMPAO) Pre-, post-MIT and 3 months post-MIT.		Pre-MIT: LH and RH hypoperfused Post-MIT: perfusion improved in LH and RH	Improvement (BDAE-SF).
Merrett et al. [34], 2018	N = 2 MIT via DVD	30 sessions in 6 weeks (5 days/week)	Age: 62–66 males Severe chronic non fluent aphasia	FMRI (n = 1) Pre and post MIT.	Speaking and 2 singing tasks (trained and untrained items).	Speaking: no changes. Singing: ↑bilateral auditory regions and RH. Perilesional LH ↓	No improvement (BDAE, Frenchay Dysartria Assessment, ABA, word retrieval/generation tasks).
Yang et al. [32], 2018	N = 6 MIT group (n = 3) vs. Conventional speech therapy group (n = 3)	32 sessions in 16 weeks (1.5 h, 2 days/week) in both therapies	Age: 47 Ischemic stroke Moderate-severe chronic non-fluent aphasia	(MRI)DTI Pre and post therapy		MIT: ↑FA in r-SLF mostly. Conventional therapy: general ↓ FA	More improvement in the MIT group (count of meaningful words with a picture description task): -MIT: 30, 285, 18 (n = 3) -non-MIT: 15, 21, 5 (n = 3)
Sandt-Koenderman et al. [21], 2016	N = 9 MIT	30 sessions in 6 weeks (5 h/week)	Subacute (n = 5): 51.2 years Chronic (n = 4): 54.0 years. Non-fluent aphasia	FMRI Pre- and post-MIT.	Auditory passive listening task	Subacute: RH and LF pre-MIT ↑RH post-MIT. Chronic: ↑LH pre-MIT, ↑LH post-MIT.	More improvement in subacute patients: -AAT: repetition, naming, spontaneous speech, auditory comprehension. -ANELT: verbal communication.
Tabei et al. [29], 2016	N = 1 MIT-Japanesse	9 days	Age: 48 male Hemorrhagic stroke Severe chronic non-fluent	FMRI Pre- and post-MIT.	Word-naming task	Correct trials: ↓ RH. Incorrect trials: ↑RH	Improvement(WAB, naming 90 words, AQ): -spontaneous speech -repetition -naming -auditory comprehension
Wan et al. [33], 2014	N = 20 MIT group (n = 11) vs. Untreated group (n = 9)	75 sessions in 15 weeks (7.5 h/week)	Age: MIT group: 55.8 9 males, 2 females Untreated group: 56.7 males Ischemic stroke Severe chronic non-fluent aphasia	MRI (DTI) Pre- and post-75 MIT sessions	_	MIT group: ↓FA in RH. Untreated group: no changes FA.	MIT group: improvement (CIU s/min (*p* < 0.001) in speech production. Untreated group: no changes CIU s/min. (ABA)
Al-Janabi et al. [27], 2014	N = 2 rTMS + MIT (Broca’s right homologue area) and sham-rTMS + MIT	3 sessions (rTMS + 40 min MIT/session) + 3 sessions (sham-rTMS + 40 min MIT/session)	Age: 65 (P.1), 49 (P.2). Males. Moderate–severe chronic non-fluent aphasia	FMRI Pre- and post-MIT.	Automatic speech task and naming/reading task	P.1: ↑LH,↓RH P.2: ↑LF and RH. No changes in naming/reading.	Phrase repetition: P.1: improvement rTMS list (*p* = 0.02). No improvement: sham-rTMS (*p* = 0.50), untreated lists (*p* = 0.91). P.2: no improvement: rTMS (*p* = 0.75), sham-rTMS (*p* = 0.43), untreated lists (*p* = 0.85). Verbal fluency: P.1: improvement (*p* = 0.09). P.2: no iprovment (*p* = 0.23)
Zipse et al. [30], 2012	N = 1 Original MIT with two additional techniques	80 sessions in 16 weeks (1.5 h, 5 sessions/week)	11 years, female Ischemic stroke Severe chronic non-fluent aphasia	FMRI and MRI(DTI) pre-, post-40 MIT, post-80 sessions and 1 year post-MIT.	FMRI: repetition of sentences with normal/intoned prosody vs. silence.	FMRI: ↑RH DTI: plasticity in r-AF and r-UF.	Improvement: -repetition: trained and untrained phrases. -picture description and semi-structured conversational (CIU s/min)
Schlaug et al. [22], 2010	N = 2 MIT	P.1: FMRI: P.2: DTI 75 sessions.		P.1: FMRI P.2: MRI (DTI) Pre and post MIT.	FMRI: speaking vs. silence (control), speaking vs. vowel production.	FMRI: ↑RH DTI: plasticity r-AF.	
Sandt-Koenderman et al. [18], 2010	N = 1 MIT	2–8 weeks (5 h/week)	Age: 25 female Ischemic stroke Severe subacute non-fluent aphasia	FMRI Pre and post	Lexical task with auditorily presented spoken or intoned words and non-words.	Pre-MIT: RH Post-MIT: more: ↑LH No differences between spoken and melodic language	Improvement: AAT -spontaneous speech: 1/5->3/5 -repetition: T = 39->T = 47 -naming:T = 39->T = 46 CIU s/minute:22.5->55
Breier et al. [23], 2009	N = 2 MIT	2 MIT blocks: 12 h Each block: 3 weeks, 2 days/week (4 sessions, 30 min)	Age: 55 (P.1), 49 (P.2). Males. Ischemic stroke Chronic mixed aphasia	MEG Pre and post each block	Action-naming test	Pre-MIT: ↑LH (P1, P2) Post-MIT: ↑ LH, ↓ RH (P1); RH and LH ↑P2).	P.1: improvement (CIU s/min > 35%) P.2: no improvement (CIU s/min).
Schlaug et al. [26], 2009	N = 6 MIT	75 sessions	Moderate–severe chronic non-fluent aphasia	MRI(DTI)Pre- and post-MIT		Plasticity in right AF.	Improvement (CIU s/min). More a patient improved->more AF fibers were detected (*r* = 0.7; *p* = 0.1).
Schlaug et al. [31], 2008	N = 2 P.1: MIT vs. P.2: SRT + MIT	P1: 75 MIT sessions (1.5 h, 5 days/week. P2: 40 SRT sessions + 75 MIT sessions (1.5 h/5 days/week)	Age: 47 (P,1), 58 (P.2). Males. Ischemic stroke. Severe chronic non-fluent aphasia	FMRI P1: pre, post 40 and post 75 MIT sessions P2: pre, post SRT, post 40 and post 75 MIT sessions	Spoken/sung bisyllabic words/phrases (experimental) and humming/phonation/silence (control).	P1: RH and LH (pre) and more ↑RH (post) P2: ↑LH, ↑ RH (pre and post SRT); more ↑RH,↓LH (postMIT)	P1: improvement. P2: more improvement after MIT than SRT. Changes %: -CIU s/min:315.90 (P.1), 301.50 (P.2) -Syllables/phrase: 261.10 (P.1), 252.50 (P.2) -Picture naming: 158.30 (P.1), 123.60 (P.2).
Belin et al. [24], 1996	N = 7 TMR	TMR during 1 month–9 years	Age: 49.7 Ischemic stroke Severe chronic non-fluent aphasia: Broca’s aphasia (n = 2) and global aphasia (n = 5).	PET Post-TMR	Rest, hearing, repetition of simple words and MIT-loaded words	Hearing vs. rest: ↑ RH Simple repetition vs. hearing: ↑RH MIT repetition vs. simple repetition: ↑LH	Improvement (*p* < 0.01) (BDAE): -expression -comprehension
Laine et al. [25],1994	N = 3 MIT		Age: 58(P.1),48(P.2), 68(P.3). Ischemic and hemorrhagic stroke Aphasia: Broca’s (P.1), mixed non-fluent (P.2), Wernicke’s (P.3)	SPECT (^99m^Tc-HM-PA0) Pre MIT	Ordinary and MIT repetition	P1: ↑ LH P2: ↑RH and LH P3: no difference	P1 and P.2: no improvement P.3: -
Christensen et al. [19], 1989	N = 1 MIT		31 years, male Severe chronic non-fluent aphasia.	SPECT (133Xe-inhalation)	Rest, ordinary repetition, and MIT	Rest: ↓LH Ordinary repetition: ↑LH and RH MIT repetition: ↑LH and greater ↑RH	

AAT: Aachen Aphasia Test. ABA: apraxia battery for adults. ANELT: Amsterdam Nijmegen Everyday Language Test. AQ: aphasia quotient. BDAE-SF: Boston Diagnostic Aphasia Examination—Short Form. BNT: Boston Naming Test. CCAT: Concise Chinese Aphasia Test. CIU: correct information units. DTI: diffusion tensor imaging. FA: fractional anisotropy. FMRI: functional magnetic resonance. LH: left hemisphere. PET: positron emission tomography. P.1: patient 1. P.2: patient 2. Pre-MIT: before Melodic Intonation Therapy. Post-MIT: after Melodic Intonation Therapy. RH: right hemisphere. r-AF: right arcuate fasciculus. r-IFL: right inferior longitudinal fasciculus. r-SLF: right superior longitudinal fasciculus. rTMS: repetitive transcranial magnetic stimulation. r-UF: right uncinate fasciculus. SPECT: single photon emission computed tomography. SRT: speech repetition therapy. TMR: thérapie mélodique et rythmique. WAB: Western Aphasia Battery. ↑: increase. ↓: decrease.

## 4. Discussion

The aim of this systematic review was to study the effects of MIT in language lateralization and neuroplastic reorganization by reviewing all the studies which included neuroimaging and/or neurophysiological techniques in non-fluent aphasia post-stroke patients. In general, the results obtained are diverse, as some studies observed a predominantly higher right hemisphere involvement, while in others, a greater left hemisphere activation was demonstrated or even a bihemispheric involvement.

### 4.1. Right Hemisphere Involvement

Firstly, according to the main hypothesis, the right hemisphere played an important role in language, since in several studies a greater right hemisphere activation was evidenced after MIT during several fMRI tasks (repetition of intoned or spoken words, passive listening or in silence) [22,29,30,31]. Therefore, the intonation and rhythm might promote a higher right hemisphere involvement than other therapies since the right hemisphere has a dominant role in the processing of spectral information, musical components, and prosody, and it is better suited for processing slowly modulated signals. In this way, probably, the slower rate of articulation and intonation facilitates this process while left hand-tapping eases auditory–motor mapping and engages a sensor–motor network that controls hand and mouth movements [7,8,9]. Moreover, MIT may also enhance neural efficiency by decreasing the cognitive load and improving language outcomes, as it is an intensive and repetitive therapy. This was evidenced in some cases: Zipse et al. [30] observed a decrease in right hemisphere activity after a few weeks of treatment in their patient, and Tabei et al. [29] found a deactivation in the right hemisphere during correct naming trials in contrast to the incorrect ones.

A few articles reported changes in different white matter after MIT therapy. Increases in the volume and number of fibers were found mainly in the right arcuate [22,26,30] and uncinate [30] fasciculus. Furthermore, in two more studies [32,33], these changes were measured by the calculation of fractional anisotropy values. In this manner, Yang et al. [32] evidenced an increase in the FA mainly in relation to the right superior longitudinal fasciculus, whereas Wan et al. [33] observed a reduction in the FA in the white matter underlying the right inferior frontal gyrus, posterior superior temporal gyrus, and the posterior cingulum. Thus, MIT might lead to changes in white matter tracts in the right hemisphere, and this discrepancy may reflect different mechanisms by which different brain regions can remodel. 

### 4.2. Left and Bilateral Hemisphere Involvement

In contrast, in several studies a greater left activation was evidenced, with a decrease in right hemisphere activity after MIT. Moreover, right hemisphere activation was found precisely in those patients who did not benefit from this therapy [23,24]. Therefore, in some cases, language recovery might be through the recruitment of the peri-lesional areas in the left hemisphere, and a greater right hemisphere activity is related to a maladaptive process and the persistence of aphasia. Nevertheless, in one of these articles, only the cases of patients who were good responders to the TMR (thérapiemélodique et rythmique) were reported, and the time between examination varied from 1 month and 9 years [24]. 

Other studies found a bilateral hemispherical involvement. For example, Martzoukou et al. [28] reported a case using SPECT after rehabilitation with MIT concerning language benefits. Christensen et al. [19] reported the same evidence comparing brain activity during rest, ordinary repetition, and MIT repetition in a patient. Nevertheless, in this case, the exact location of the lesions was not known, nor whether MIT therapy was received after the measurement, so it is not possible to relate it with clinical benefits. In addition, in the study of Merrett et al. [34], one patient received MIT therapy via DVD instead of from a therapist, which is the usual way. However, this patient did not improve his speech outcomes since the therapy was not well adapted to his necessities since he could not accurately produce any words or phrases of the MIT items or those used during the fMRI task. Nonetheless, after the end of the study, he demonstrated considerable improvement after he continued the MIT with a therapist.

### 4.3. Clinical Characteristics of the Patients

On the other hand, although most of the participants had chronic aphasia, two studies included subacute aphasic patients, though with heterogeneous results: in one subacute aphasic patient, a left perilesional hemisphere activation was shown on the fMRI [18]; in contrast, in another study, a right lateralization was observed in subacute aphasic patients and a left lateralization was observed in the chronic patients [21]. After a stroke, in the acute phase, there is just a little activation of the left perilesional structures, but in the subacute phase, there is a strong bilateral activation in the language network, mostly in the right hemisphere. Finally, in the chronic phase, language activation returns to a normal pattern, with a higher activation in the left areas [35]. Nonetheless, in patients with extensive left hemisphere lesions, intensive right hemisphere activation is necessary to compensate the burden. For these reasons, the participant in the first study probably had the left hemisphere less damaged. 

Finally, some of the participants included did not benefit from this therapy. A good response to MIT therapy was associated with lesions in Broca’s area, but with no significant lesion in Wernicke’s area or in the right hemisphere. Moreover, patients should have their receptive language intact and be emotionally stable. In contrast, a poor response was associated with lesions in Wernicke’s area or both hemispheres [5,6]. Thus, some of the participants probably did not benefit from this therapy since they did not match these criteria [23,25,34]. Nevertheless, the participant of Martzoukou et al. [28] benefited from MIT, despite having damage to Wernicke’s area and bilateral hemispheric lesions. Furthermore, five of the patients included in the study of Belin et al. [24] responded well to TMR (thérapie mélodique et rythmique), although they had global aphasia. 

### 4.4. Limitations

This review has some limitations. First, the small sample of patients in each study and the heterogeneity, since participants of all ages, either sex, and with all types of aphasia post-stroke were included. Moreover, neuroimaging/neurophysiological techniques varied between the studies, so, in some cases, white matter tracts were evaluated, while in others, the objective was to observe language lateralization with fMRI, SPECT, or PET during different tasks, such as MIT repetition, ordinary repetition, passive listening, naming/reading, etc. In some cases, MIT differed among the studies because the therapy was performed in different languages, using technological means such as a DVD instead of a therapist [34] and with other versions, such as TMR [24]. In addition, in two studies, language lateralization was just assessed during MIT repetition in contrast to ordinary repetition, so we cannot relate neuroimaging results with clinical benefit [19,25]. In contrast, in one study, only cerebral blood flow was measured during hearing, ordinary repetition, and MIT repetition in patients who were previously good responders to TMR [24]. Furthermore, only four articles included a control group in order to compare MIT with other conventional language therapies or a non-treated group of patients. Finally, a sample of the participants did not match the criteria for good candidacy for MIT therapy, so their results are difficult to interpret.

## 5. Conclusions

MIT is a good option to rehabilitate patients with non-fluent aphasia post-stroke, since, in many studies, patients showed improvement in language outcomes. Different neurobiological mechanisms are described to explain how MIT leads to recovering language function. On the one hand, some studies support right hemisphere involvement, which was the original MIT idea, whereas, in other cases, a greater left hemisphere activity was evidenced, or even a bilateral involvement. For this reason, we could not come to any general conclusion about one specific mechanism by which MIT acts on brain plasticity. Probably, hemispherical involvement would depend on multiple individual factors: in patients who had the left hemisphere less damaged, this therapy may lead to a recruitment of the left perilesional areas to recover language function. However, in cases with very extensive lesions in the left hemisphere, the right hemisphere might attempt to assume language function. Accordingly, more studies with large samples of patients, as well as studies with a control group, since the majority of studies only include a series of cases, are necessary in order to extrapolate the conclusions for the general population. 

## Data Availability

Not applicable.

## References

[1] Cichon N., Wlodarczyk L., Saluk-Bijak J., Bijak M., Redlicka J., Gorniak L., Miller E. (2021). Novel Advances to Post-Stroke Aphasia Pharmacology and Rehabilitation. J. Clin. Med..

[2] Berthier M.L., Pulvermüller F., Dávila G., Casares N.G., Gutiérrez A. (2011). Drug therapy of post-stroke aphasia: A review of current evidence. Neuropsychol. Rev..

[3] Albert M.L., Sparks R.W., Helm N.A. (1973). Melodic Intonation Therapy for Aphasia. Arch. Neurol..

[4] Sparks R., Helm N., Albert M. (1974). Aphasia Rehabilitation Resulting from Melodic Intonation Therapy. Cortex.

[5] Helm-Estabrooks N., Albert M.L. (2004). Melodic Intonation Therapy: Manual of Aphasia and Aphasia Therapy.

[6] Naeser M.A., Helm-Estabrooks N. (1985). CT Scan Lesion Localization and Response to Melodic Intonation Therapy with Nonfluent Aphasia Cases. Cortex.

[7] Zumbansen A., Peretz I., Hébert S. (2014). Melodic intonation therapy: Back to basics for future research. Front. Neurol..

[8] Merrett D.L., Peretz I., Wilson S.J. (2014). Neurobiological, cognitive, and emotional mechanisms in melodic intonation therapy. Front. Hum. Neurosci..

[9] Norton A., Zipse L., Marchina S., Schlaug G. (2009). Melodic intonation therapy: Shared insights on how it is done and why it might help. Ann. N. Y. Acad. Sci..

[10] Van Eeckhout P., Bhatt P. (1984). Rythme, intonation, accentuation: La rééducation des aphasies non-fluentes sévères. Rééduc. Orthophonique.

[11] Haro-Martínez A., Pérez-Araujo C.M., Sanchez-Caro J.M., Fuentes B., Díez-Tejedor E. (2021). Melodic Intonation Therapy for Post-stroke Non-fluent Aphasia: Systematic Review and Meta-Analysis. Front. Neurol. Front. Media SA.

[12] Page M.J., McKenzie J.E., Bossuyt P.M., Boutron I., Hoffmann T.C., Mulrow C.D., Shamserr L., Tezlaff J.M., Akl E.A., Brennan S.E. (2021). The PRISMA 2020 statement: An updated guideline for reporting systematic reviews. BMJ (Clin. Res. Ed.).

[13] Van de Sandt-Koenderman W.M.E., Mendez C.E., Bechan M.A.H., Van der Meulen I.A.C., Ribbers G.M., Visch-Brink E.G., Smits M. Brain plasticity in aphasia Functional MRI pre and post Melodic Intonation Therapy (MIT). Proceedings of the International Brain Injury Association’s Ninth World Congress on Brain Injury.

[14] Tseng C.E., Yang F.P.G., Lin C.P. Melodic Intonation Therapy in Stroke Patients with Aphasia: A DTI Study. Proceedings of the Annual Conference of the Asia Pacific Stroke Organization (APSO).

[15] Vines B.W., Norton A.C., Schalug G. (2011). Non-invasive brain stimulation enhances the effects of melodic intonation therapy. Front. Psychol..

[16] Altenmüller E., Schlaug G. (2015). Apollo’s gift: New aspects of neurologic music therapy. Prog. Brain Res..

[17] Fridriksson J., Hubbard H.I., Hudspeth S.G., Holland A.L., Bohilha L., Fromm D., Roden C. (2012). Speech entrainment enables patients with Broca’s aphasia to produce fluent speech. Brain.

[18] Van de Sandt-Koenderman M., Smits M., Van der Meulen I., Visch-Brink E., Van der Lugt A., Ribbers G. (2010). A case study of melodic intonation therapy (MIT) in the subacute stage of aphasia: Early re-reactivation of left hemisphere structures. Procedia. Soc. Behav. Sci..

[19] Christensen A.L., Jensen L.R. (1989). Luria’s Neuropsychological and Neurolinguistic Testing. J. Neurolinguistics.

[20] Maher C.G., Sherrington C., Herbert R.D., Moseley A.M., Elkins M. (2003). Reliability of the PEDro scale for rating quality of randomized controlled trials. Phys. Ter..

[21] Van de Sandt-Koenderman M.W.M.E., Mendez Orellana C.P., Van der Meulen I., Smits M., Ribbers G.M. (2018). Language lateralisation after Melodic Intonation Therapy: An fMRI study in subacute and chronic aphasia. Aphasiology.

[22] Schlaug G., Norton A., Marchina S., Zipse L., Wan C.Y. (2010). From singing to speaking facilitating recovery from nonfluent aphasia. Future Neurol..

[23] Breier J.I., Randle S., Maher L.M., Papanicolau A.C. (2010). Changes in maps of language activity activation following melodic intonation therapy using magnetoencephalography: Two case studies. J. Clin. Exp. Neuropsychol..

[24] Belin P., Van Eeckhout P., Zilbovicius M., Remy P., François C., Guillaume S., Chain F., Rancurel G., Samson Y. (1996). Recovery from nonfluent aphasia after melodic intonation therapy: A PET study. Neurology.

[25] Laine M., Tuomainen J., Ahonen A. (1994). Changes in hemispheric brain perfusion elicited by Melodic Intonation Therapy: A preliminary experiment with single photon emission computed tomography (S PECT). Scand. J. Log. Phon..

[26] Schlaug G., Marchina S., Norton A. (2009). Evidence for plasticity in white-matter tracts of patients with chronic broca’s aphasia undergoing intense intonation-based speech therapy. Ann. N. Y. Acad. Sci..

[27] Al-Janabi S., Nickels L.A., Sowman P.F., Burianová H., Merrett D.L., Thompson W.F. (2014). Augmenting melodic intonation therapy with non-invasive brain stimulation to treat impaired left-hemisphere function: Two case studies. Front. Psychol..

[28] Martzoukou M., Nousia A., Nasios G., Tsiouris S. (2021). Adaptation of Melodic Intonation Therapy to Greek: A Clinical Study in Broca’s Aphasia with Brain Perfusion SPECT Validation. Front. Aging. Neurosci..

[29] Tabei K.I., Satoh M., Nakano C., Iton A., Shimoji Y., Kida H., Sakuma H., Tomimoto H. (2016). Improved neural processing efficiency in a chronic aphasia patient following melodic intonation therapy: A neuropsychological and functional MRI study. Front. Neurol..

[30] Zipse L., Norton A., Marchina S., Schalug G. (2012). When right is all that is left: Plasticity of right-hemisphere tracts in a young aphasic patient. Ann. N. Y. Acad. Sci..

[31] Schlaug G., Marchina S., Norton A. (2008). From Singing to speaking: Why singing may lead to recovery of expresive language function in patients with broca’s aphasia. Music Percept..

[32] Yang F.P.G., Wang N.Y.H., Lin C.P., Chiang T.M., Lin C.M., Lai Y.T. (2019). Change of neuronal pathways in Chinese speakers with non-fluent aphasia after therapy. Lingua.

[33] Wan C.Y., Zheng X., Marchina S., Norton A., Schalug G. (2014). Intensive therapy induces contralateral white matter changes in chronic stroke patients with Broca’s aphasia. Brain Lang..

[34] Merrett D.L., Tailby C., Jackson G.D., Wilson S.J. (2019). Perspectives from case studies in obtaining evidence for music interventions in aphasia. Aphasiology.

[35] Saur D., Lange R., Baumgaertner A., Schraknepper V., Wilmes K., Rijntjes M., Weiller C. (2006). Dynamics of language reorganization after stroke. Brain.

